# The relationship between work and health in unpaid carers: part II of a scoping review and new model

**DOI:** 10.1186/s12889-026-28522-7

**Published:** 2026-07-28

**Authors:** Clarissa Giebel, Leah Shinn, Sheena Johnson, Gill Windle, Joan Carton, Jennie Popay, Debora Price, William Whittaker, Mark Gabbay

**Affiliations:** 1 Department of Primary Care and Mental Health, University of Liverpool, Liverpool, UK; 2 NIHR Applied Research Collaboration North West Coast, Liverpool, UK; 3 Alliance Manchester Business School, University of Manchester, Manchester, UK; 4 School of Health Sciences, Bangor University, Bangor, UK; 5 School of Health Research, Lancaster University, Lancaster, UK; 6 Department of Sociology, University of Manchester, Manchester, UK; 7 Department of Public Health, University of Liverpool, Policy & Systems, Liverpool, UK

**Keywords:** Carers, Workforce, Social care, Mental health, Employment

## Abstract

**Background:**

Unpaid carers can struggle to manage caring duties alongside paid employment, often consequently changing their work role, reducing hours or leaving employment. However, less is known about how working whilst delivering unpaid care is linked to physical and/or mental health outcomes. Thus, the aim of this scoping review was to explore and compare the existing published evidence on the relationship between paid work and health in unpaid carers for dependants aged 18 + .

**Methods:**

Four online databases were searched in October 2025 for published research on the relationship between paid work and health in unpaid carers, in English or German, and published between 2000 and 2025. Papers were not restricted by methodological approach. Three researchers reviewed titles and abstracts, then two research team members screened full texts. Any disagreements were discussed and settled within the wider research team.

**Results:**

Fifty-eight papers from 21 countries met inclusion criteria for this review. Evidence was categorised into three levels, informing a new model on the relationship between paid work and health in unpaid carers – the *Carers’ Work and Health Support Model*: Personal; workplace; and system and society factors. The relationship between paid work and health in unpaid carers is complex, with some carers benefitting from working longer hours and others being negatively affected. The majority of evidence reported negative impacts on mental health when having unpaid caring duties whilst in employment, with supportive management and colleagues critical for wellbeing.

**Conclusions:**

This novel scoping review is the first to present a co-produced and systematically evidence-derived model on the relationship between paid work and health in unpaid carers. Work-place based interventions need to be designed and tested to improve employee well-being and maintain unpaid carers in the paid workforce.

## Introduction

Unpaid carers provide a pivotal support system that often fails to get recognised, whilst effectively taking pressure off the paid social care workforce (The Lancet Public [75]. The most recent UK Census 2021 data for example identified 5.8 million unpaid carers, with three million providing unpaid care whilst in employment, and 2.6 million having left employment due to caring [10].

Over time, working age (aged 18 +) unpaid carers experience negative mental health outcomes linked to caregiving [21], with an abundance of evidence pointing to high levels of stress and burnout [27, 35] and generally poorer mental health and well-being among unpaid carers with higher levels of caring hours [70].

Having to quit employment as an unpaid carer is suggested to be a strong predictor of poverty [10]. Furthermore, unpaid carers leaving employment have a negative impact on the national economy, with estimates from 2016 indicating the costs in England of unpaid carers leaving employment at £2.9 billion/year, including substantial social security benefits: income support, housing benefit, and carers allowance [61]. Evidence from the English Longitudinal Survey of Ageing reveals that male and female carers providing 10 h or more of unpaid care are less likely to be in employment at the next wave of data collection. In contrast, female carers providing less than 10 h of care per week, were significantly more likely to still be in employment than those without caring duties [40]. It has been suggested that this employment may be protective against gendered expectations of caring roles [62]. Thus, being mindful of the intensity of caring provided by a substantial proportion of the general workforce and ensuring adequate support mechanisms are available for carers to enable them to remain in employment are critical. However, unfortunately for many, these are not available.

Considering the high levels of working age carers reducing their working hours or leaving employment altogether [9], it is important to understand the relationship between paid work and health in this growing population. Research on unpaid carers aged 16 and above across the UK has shown that lower-qualified carers providing 10 h or more of unpaid care a week, were significantly less likely to be in employment whilst caring, had less income, and had poorer physical and mental health outcomes than higher-qualified carers [8]. Linked to this, evidence from Japan highlights that compared to employees without caring duties, those caring for an elderly relative were at higher risk for depression [34]. However, the relationship between paid work and health is complex, nuanced and depends on various factors, which need to be comprehensively synthesised in order to understand these intricate relationships and develop targeted guidance and support.

Our searches did not identify any structured review to date summarising existing research evidence on the relationship between paid work and health among unpaid carers. One review has explored evidence into the challenges of working when caring [69]. Within that scoping review, which included 92 papers, some studies focused on carer’s health. However, the authors did not summarise individual studies on this subject, providing only a top-level overview of some studies evidencing a lack of sleep when providing unpaid care leading to exhaustion and thus impacting on work and sick leave. Thus, the aim of this scoping review was to explore the evidence on the relationship between paid work and health in the unpaid care workforce. This scoping review is part II of a two-part scoping review on the relationship between paid work and health in the wider care workforce, with part I focusing on paid carers ([26] submitted) . Findings from this review can identify areas for future research with direct impact on retaining employees undertaking unpaid care while in the workforce, whilst supporting their health and wellbeing.

## Methods

A five-stage methodological framework process (Arksey & O’Malley, 2005) was implemented for the current scoping review, due to its conceptually broad nature. A scoping review was the most suitable given the focus on including studies using any research design, without a specific research question on outcomes, as we intentionally planned on reviewing the evidence broadly. Thus, we conducted the five steps of the framework, including (1) identifying the research question; (2) identifying relevant studies; (3) selecting relevant studies; (4) extracting and charting the data; and (5) collating and synthesising the evidence. Additionally, to further strengthen the model development, we consulted un/paid carers to consolidate the findings emerging from the evidence base and ground them in the lived experiences of unpaid and paid carers. This additional step is recommended by Arksey and O’Malley (2005). Carer consultation did not influence the screening, selection, or data extraction process of the review though. By taking an exploratory approach, we aimed to present a thorough understanding of previous literature concerning the relationships between health outcomes and employment in those who provide unpaid care.

### Search strategy

The following four databases were searched in October 2025: PubMed, CINAHL Ultimate, Scopus and Apa PsycInfo. The principal investigator (CG) and one research team member (LS) collaboratively developed a list of search terms. These were piloted and altered if necessary. The following search terms were used:

(caregiver* OR carer* OR “care assistant*” OR “adult social care” OR “unpaid support”) AND (employment OR work) AND (health OR wellbeing OR “mental health” OR burnout OR stress) NOT (childcare OR child* OR pediatr* OR nurse* OR doctor*).

Subject headings were combined where applicable to improve accuracy of retrieved papers. Results were filtered to present studies written in the English or German language, and restricted to only those published between 2000 and 2025. All records were retrieved (n = 2,677) in their respective format and then uploaded to the online screening tool Rayyan, where 566 duplicate papers were removed. Further papers were identified by secondary and hand searches (two for each of these reviews).

### Inclusion and exclusion criteria

In order to ensure consistency with the framework’s emphasis on capturing evidence breadth, studies were not limited by methodology: quantitative, qualitative and mixed method studies were included. Research was included if it detailed a primary focus on the relationships between health and paid work in unpaid carers. Included studies were not restricted by the dependant’s condition or type of care provided. Research findings which solely investigated the care of a child or younger person were excluded. Publications reporting only one of the outcomes, either health or paid work, were also excluded. Papers which investigated these issues among paid carer workforces were included in our parallel scoping review.

### Study selection

**Fig. 1:**
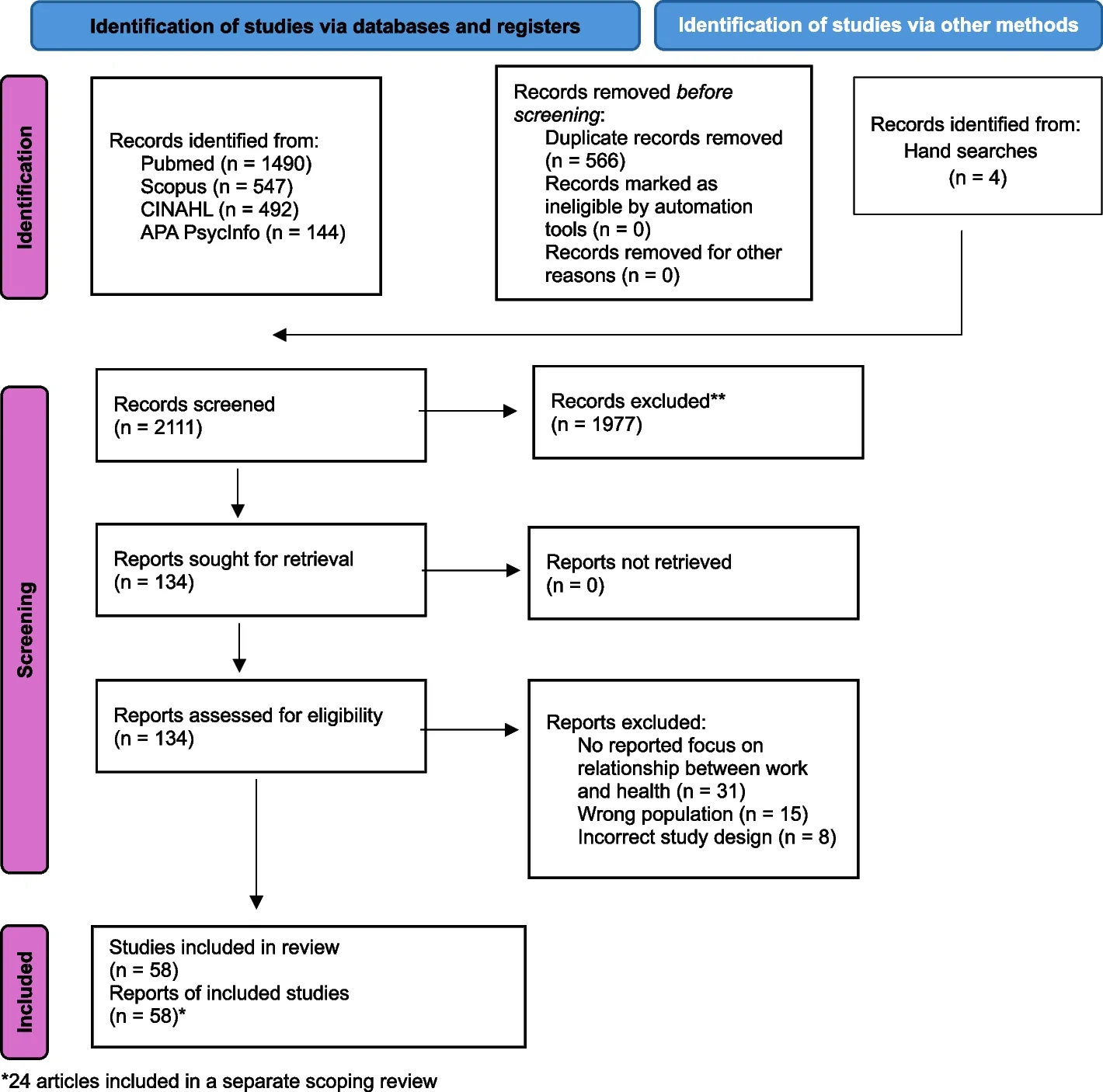
PRISMA flowchart

Each title and abstract of retrieved records after all duplicates had been removed were screened by two researchers, using Rayyan, against inclusion criteria. We thus excluded 1,977 articles which did not meet inclusion criteria in Stage 1. This task was shared among three research team members (CG, LS, GW), ensuring that each title was assessed by two reviewers. The 134 remaining publications were then read in full text in Stage 2, independently by two researchers (CG, LS) and only articles that met the full inclusion criteria were included in this current review. Any discrepancies at stage 1 or 2 were resolved in discussion with the wider research team, which also included three public and care provider stakeholders. A total of 58 articles were included in this current review. See Fig. 1 for PRISMA flowchart.

### Data extraction and synthesis

The following data were extracted by one researcher (LS): author, year, country, methodological approach, sample, and relationship between paid work and health. Narrative synthesis was used to synthesise results, discussed with the wider research team. Descriptive summaries were developed to record key characteristics of each paper, providing a clear overview of evidence distribution. Concise summaries of each text were then produced collaboratively by two reviewers (CG, LS) and organised into themes to summarise findings within Table 1. Similarities and differences between results were considered thematically in order to identify patterns from the findings and map their relationship, identifying impacts on both physical and mental health. A comprehensive understanding was encouraged from this approach and allowed identification of the three levels of evidence – *personal, work-related and system and societal factors*.

**Table 1:** Key characteristics of included studies

Author (year)	Country	Quantitative/qualitative/mixed methods	Methodology	Sample	Relationship between work and health	Relationship with physical health	Relationship with mental health
[1]	USA	Quantitative	Secondary analysis of longitudinal survey data—baseline, 4 and 12 month follow up (2009–2014)	188 unpaid carers (head/neck cancer and undergoing treatment)	• Higher psychological distress when reduced work hours or exited workforce • Those who had an increase in employment reported decreased distress		✓
[2]	New Zealand	Quantitative	Secondary analysis of longitudinal cohort data – (Health, Work and Retirement survey waves 2010 and 2014)	1036 working individuals aged 55 + 157 providing unpaid care	• No significant differences for physical health over time • Poorer mental health, and higher depression score, in working carers vs. non-caring workers	✓	✓
[3]	Australia	Quantitative	Cross-sectional data derived from Australian Bureau of Statistics Disability, Ageing and Carers survey 1998	644 employed unpaid carers	• No relationship between working more hours and stress • But caring for someone with a mental disability and working more hours is linked to lower stress		✓
[5]	Netherlands	Qualitative	First round of semi-structured interviews from longitudinal study	18 unpaid family carers (working more than 12 h a week)	• Poor work-care balance associated with feeling listless, irritable and moody • Increased headaches, panic attacks and antidepressant use • Reducing work hours have mixed effects on wellbeing, depending on whether it improves providing unpaid care	✓	✓
[6]	USA	Quantitative	Health and Retirement study (2002–2020) Structured interviews collecting survey data (over 4 years)	103 employed spousal carers with cancer 515 employed spousal carer of another condition	• After adjusting for covariates (income decrease, debt increase and stopping work) greater percentage of cancer caregivers reported adverse mental health, than other carers • However results were mixed and non-significant		✓
[7]	USA	Quantitative	Secondary analysis of Health and Retirement survey 2004–2018 longitudinal cohort data	13,335 in sample 2398 combining paid work and parental caregiving	• Working while parental caring increased depressive symptoms, compared to only working • Working while parental caring associated with lower depressive symptoms, compared to only caring • White women more likely to experience poorer mental health than white men when working and caring		✓
[8]	UK	Quantitative	Survey data UK Household Longitudinal Study (wave 9, 2017/19 and wave 10, 2018/20)	23,586 non-carers and 2,349 carers who provided 10 + hours of care 23,586 non-carers and 1,768 co-resident carers	• Providing care for 10 + hours a week associated with lower earnings, lower likelihood of being in employment and poorer physical/mental health • Lower qualified carers who care for ten or more hours six times less likely to be in paid employment and had significantly lower mental/physical health scores than highly qualified carers	✓	✓
[11]	China	Quantitative	Secondary analysis of longitudinal cohort data, 2000–2015 China Health and Nutrition Survey	10,828 female full-time workers Reduced analytic sample with health data (n = 6,158)	• Negative impact on health when juggling caregiving and employment • 20 + hours parent/in-law care and working 50 h + a week led to poorest health • Increased risk of chronic disease and being under/overweight	✓	
[12]	UK	Quantitative	Cross-sectional survey data from IDEAL study Time 1 (2014–2016)	1188 family carers (dementia)	• Overall, working carers had better life quality than non-working carers. Matched by age, working carers had better wellbeing and quality of life than non-working carers • Differences influenced by relative stress, role captivity and carer-dyad relationship	✓	✓
[13]	Spain	Quantitative	Cross-sectional structured interview and self-administered survey data	83 employed unpaid carers of older adults	• 23%—difficulties combining roles • Role captivity and role overload predict negative affect • Worry/strain predicted by hours of caregiving and job-caregiving conflicts • Perceived job conflicts influenced emotional state more than actual job conflicts		✓
[16]	UK	Quantitative	Longitudinal and biomarker data—Whitehall II cohort study (1991–2004)	7007 British civil servants, some providing unpaid care	• High caregiving burden predicted higher allostatic load, highest effect in those who had high job strain • However, the interaction was non-significant • In non-carers and participants with low carer burden, job strain was minimal	✓	✓
[15]	UK	Quantitative	Longitudinal data from Whitehall II cohort study questionnaires (1991–1999)	5419 British civil service workers, some became unpaid carers during data collection	• Carers with low job demands more likely to quit smoking than non-carers • Carers with high social support at work more likely to quit smoking • Becoming a carer and low decision altitude at work increased alcohol use	✓	
[17]	UK (Northern Ireland)	Quantitative	Longitudinal national survey(s), including subjective/objective measures of mental ill-health (2001–2011)	378,365 from large dataset	• Part-time and non-employed high-intensity caregivers were more likely to be prescribed antidepressants • Women under 50, high care hours and non-employed/part time at high risk of mental ill health		✓
[18]	China	Quantitative	Survey data from Chinese Health and Retirement Longitudinal study (2011–2018)	20,213 from large dataset	• Working while providing spousal daily-living care exacerbates depression • Full time work and instrumental activities of daily living (IADL) assistance increases depression in women • Providing IADL assistance when unemployed reduces depression		✓
[19]	New Zealand	Qualitative	Semi-structured interviews	4 double duty female carers with formal and unpaid roles	• Own health put on a back burner • Juggling multiple care roles can result in stress, anxiety, pressure and conflict	✓	✓
[20]	Canada	Quantitative	Secondary analysis of cross-sectional survey data—2001 Canadian National Work, Family and Lifestyle study	(n) married individuals with eldercare responsibilities from a large data set (overall n = 31,517)	• Highest levels of physical and emotional strain in carer-employees whose dependant lives with them • When living separately, whether the dependant lives near the carer, or elsewhere, affects physical but not emotional strain	✓	✓
[22]	UK and Belgium	Quantitative	Cross-sectional national surveys—2001 British census (5% sample) and 2001 Belgian (full) census	1,361,222 Britons (14% unpaid carer) 4,368,637 Belgians (9% unpaid carer)	• Employment status influenced the association between unpaid caregiving and health in both countries • Working and caring linked to worse health outcomes than non-care	✓	✓
[24]	USA	Quantitative	Cross-sectional, correlational survey design	183 unpaid carers (cancer)	• Females combining paid work and unpaid caring reported more exhaustion vs males • Working females also more likely to provide instrumental care than men who did or did not work	✓	
[25]	USA	Mixed methods	Cross-sectional, online survey data A follow-up focus group interview with unpaid carers (n = 45)	880 employees from a large healthcare plan employer (n = 370) unpaid caregivers	• Hourly employees—poorer health than salaried • Greater caregiving-work conflict associated with poorer health • Caregiving-work conflict mediated association between burden and health • Themes added context, challenging to balance work and care	✓	✓
[28]	USA	Quantitative	Cross-sectional survey data, collected either online or via telephone	95 unpaid carers	• Leaving employment due to caring—higher financial strain/poorer mental health • No similar effect on physical health		✓
[29]	Netherlands	Quantitative	Secondary analysis of cross-sectional survey data from first wave of NIDI Pension Panel study (2015)	1651 employed unpaid carers (680 organisations)	• Perceived access to phased retirement lowers caregiving burden/stress • Perceived access to workplace medical examinations—lower burden/stress		✓
[30]	Norway	Quantitative	Cross-sectional data from Life-course, Generations and Gender study (2007–2008)	Taken from larger study (n = 11,047) 556 unpaid carers	• Caregiving and wellbeing relationship unaffected by employment in men • Out-of-household care unrelated to wellbeing across genders, regardless of employment • Caring for a partner/resident parent has adverse psychological effects among women who do not work full time		✓
[32]	USA	Quantitative	Secondary, cross-sectional data analysis using Detroit Research on Cancer Survivors cohort survey (2019)	202 employed unpaid carers (cancer survivors)	• 38% of carers struggle to balance work and care, linked to depression/anxiety • Anxiety scores 86% higher and depression 140% higher in carers who reported difficulty vs no difficulty • High financial burden was associated with anxiety in comparison to low		✓
[33]	USA	Mixed methods	Cross-sectional Surveys Semi-structured interviews	40 employed unpaid carers (Parkinson’s Disease)	• Quan: Supervisor support moderately, but coworker support significantly associated with work-life balance. High burden/stress • Qual: Poor work-life balance, physical and emotional stress. High burden impacts work but flexibility helps		✓
[34]	Japan	Quantitative	Cross-sectional Self-administered questionnaires	722 employed participants, some caring for an older relative (n = 112)	• Caring for an older relative significant risk factor for depression in employees		✓
[37]	Austria	Quantitative	Cross-sectional, online survey Part four of a larger piece of research	75 current or previous unpaid carers of a relative, while working within a university	• One third concealed carer identity from employer out of fear/shame • Majority described combination of care and work as bad or very bad • Significant correlation between burnout, employment duration and roles	✓	✓
[38]	Australia	Quantitative	Longitudinal—Household Income and Labour Dynamics in Australia – data before caregiving, 2 years and 4 years after	424 unpaid carers of which 57% were employed 424 propensity score-matched non carers	• High intensity caregiving females, working 35 h a week at baseline, had a decline in physical functioning and mental health at two years • High intensity caregiving males who were not working had improved physical functioning at two years, but poorer mental health at four years	✓	✓
[39]	USA	Quantitative	Cross-sectional survey data	583 female, full time employed, older adult unpaid carers Missing data reduced sample to 539	• Work interfering with care associated with less positive affect/more negative affect • Caregiving interfering with work not associated with either • Perceived financial need moderated relationship between work-caregiving conflict and psychological wellbeing		✓
[41]	USA	Quantitative	Secondary analysis of cross-sectional survey data - Family Function, Stroke Recovery, and Caregiver Outcomes study	132 family carers of stroke survivors	• Depressive symptoms similar in working and nonworking carers, but working carers more likely to have depression • Physical health poorer in non-working carers	✓	✓
[42]	USA	Quantitative	Cross-sectional secondary analysis: National Study of Caregiving and National Health and Aging Trends Study (2011, 2015 and 2017 waves)	3039 unpaid carer observations	• Intensive caregiving (> 80 h a month) associated with higher levels of anxiety/depression; however, work does not independently impact this		✓
[43]	USA	Quantitative	Secondary analysis of cross-sectional data – wave 1 of Health and Retirement study (1992)	527 (retired, non-retired, unpaid carers, non-carers)	• Employment status influenced association between carer status and depression, but not overall wellbeing • Increase in caregiving hours had negative impacts on mental wellbeing in those employed, compared to those who gave fewer hours		✓
[46]	Canada	Quantitative	Secondary analysis of cross-sectional data from 2012 Canada General Social Survey Cycle 26: caregiving and care receiving	1696 employed family carers	• Employment adjustments (i.e. reducing hours) significantly associated with worse self-rated mental health and higher stress • Family-to-work conflict mediates employment and mental health		✓
[45]	USA	Quantitative	Observational, cross-sectional secondary analysis of National Survey of Midlife Development data (2004–2006)	67 employed carers (disability) who had a supervisor at work	• When caregiving demands/family strains were high, higher family-to-work conflict reported • High family to work conflict associated with increased depressive symptoms and decreased life satisfaction, but only when perceived supervisor support was low • Level of supervisor support moderated this indirect impact caregiving had on life satisfaction		✓
[47]	Canada	Quantitative	Using social role theory, cross-sectional survey data was derived from the 2012 Canada General Social Survey Cycle 26: caregiving and care receiving	2426 employed unpaid carers	• Women who combine unpaid care and employment—poorer mental health than men • Greater employment adjustment and family-to-work conflict experienced in women • Workplace support (depending on how much available) could significantly moderate employment adjustment and poorer mental health		✓
[48]	USA	Quantitative	Longitudinal survey data: In person interview at baseline Structured telephone interviews at 6- and 12-months post baseline	184 unpaid carers (co-residing with a relative with dementia)	• Role overload, from combining caring and paid work, seen as a stressor • Caregivers with greater financial strain at baseline had higher levels of role overload and increasing role captivity over time • Carers who were never employed had lower levels of role overload than employed		✓
[49]	USA	Quantitative	Secondary, cross-sectional analysis of 2009 Fifth National Survey of Older Americans Act program	922 unpaid carers of traditional working age adults	• Quitting or retiring from work early due to caregiving associated with greater stress • Work interference significantly associated with higher levels of emotional stress associated with caregiving demands		✓
[50]	Singapore	Qualitative	2010 paper, originally 75 semi structured interviews	30 family carers 15 live-in carers (in separate review)	• Conflict, stress and mental exhaustion felt when balancing work and caregiving • Many depended on foreign domestic workers to cope, but cost and communication were challenging • Using a FDW was mentally beneficial		✓
[52]	Puerto Rico, Mexico and Colombia	Quantitative	Cross-sectional self-administered questionnaires	1194 Hispanic unpaid carers of older adults	• Significant positive correlation between employment status and depression		✓
[54]	USA	Quantitative	Cross-sectional survey data	187 employed carers of an Allo-HSCT patient	• Average score indicated clinical depression and mild anxiety in employed caregivers • In unadjusted comparisons, employment reduction associated with increased depression • In adjusted comparisons, reducing hours associated with increased depression and anxiety		✓
[56]	Australia	Quantitative	Cross-sectional cohort—2011–12 Life Histories and Health survey data A follow up structured telephone interview	1261 baby boomers, some providing unpaid care (n = 153)	• Carers had poorer health than non-carers • Working carers had fewer mobility problems and better health vs non-working	✓	
[57]	21 high income, European countries	Quantitative	Cross-sectional survey data Seventh round of the European Social Survey 2014	Sub sample of 11,177, 5743 employed unpaid carers	• 16.5% of non-working carers reported depression compared to 9.6% of working • Not working – more likely to report issues with heart/circulation, blood pressure, back or neck pain and muscular/joint pain in feet or hands	✓	✓
[58]	Japan	Quantitative	Cross sectional survey	171 unpaid carers, 32.7% of which were employed	• Taking care leave from work significantly associated with depression • A change in employment condition –becoming a part time worker—had a negative association with depression		✓
[59]	South Korea	Quantitative	Cross-sectional survey data - Korean Working Conditions survey, sixth round	33,063 employees, some of which provided unpaid care (n = 1541)	• Long working hours combined with caregiving activities associated with poor psychological wellbeing		✓
[60]	USA	Quantitative	Cross-sectional data from: National Study of Caregiving Round 7 (2017) Time diaries	2136 unpaid carers 1005 were employed	• “Day off” carers, providing only light care prior to working, reported best wellbeing • “Late shifts” carers had poorest wellbeing in comparison to other carers • “Care after work” had similar wellbeing to “late shifts” • “Balancing act” carers had similar wellbeing to “Day off” • “Care after overwork” had lower wellbeing than the “Day off” group		✓
[63]	Sweden	Quantitative	Swedish Longitudinal Occupational Survey of Health (2010, 2012, 2014, 2016)	21,604 employees, some providing unpaid care	• Providing < 5 h of care per week, and providing more than 5 h care per week, compared to no care, associated with more sleep disturbance • Cessation of caring statistically linked to reductions in sleep disturbances	✓	
[64]	Canada	Quantitative	Cross-sectional—Canadian national survey—Balancing Work, Family, and Caregiving National Survey 2015–2016	Unpaid carers of an older adult (n = 1839) some of which lived with dementia (n = 666)	• Higher dementia demands meant greater stress, depressed mood, self-efficacy, burden and role overload • Also meant higher job-caregiving conflict, absenteeism and days off due to ill health		✓
[67]	Germany	Quantitative	Cross-sectional survey data waves from German Socioeconomic panel (2002–2018)	3053 unpaid carers	• Full time employed – significantly better mental health than unemployed/marginally employed • Part time employed reported slightly better mental health vs unemployed/marginally employed • Moderating effect of net household income, association is weaker as income increases		✓
[68]	Italy	Quantitative	Longitudinal, observational data from UP-TECH project	146 unpaid carers for older people with Alzheimer’s disease	• Non-working carers reported higher levels of burden than other carers • No differences in working situations concerning mental health • No interference to working hours—better physical health than those who were forced to reduce hours	✓	✓
[71]	Sweden	Quantitative	Swedish Time Use Survey (time diary) data, 2000–01 and 2010–11	Individuals aged 45–74 (n = 6689), 1197 of which were unpaid caregivers	• Employed women, particularly when they combined unpaid caregiving, were significantly more likely to report stress than men		✓
[72]	USA	Qualitative	Semi-structured interviews	30 unpaid carers (cancer)	• Employment could provide respite • Some had increased job-related stress and inability to concentrate • Work support reduced emotional strain		✓
[73]	USA	Quantitative	Secondary analysis of cross-sectional survey data – AARP and National Alliance for Caregiving population-based study (2015)	369 full-time employed carers of adults aged over 50	• Greater caregiving-to-work conflict—more physical, emotional and financial strain • Disclosing caregiving to employers linked to increased emotional/physical strain • Workplace policies did not significantly buffer caregiving-work-conflict or strain	✓	✓
[74]	USA	Quantitative	Secondary analysis of cross-sectional data Self-administered questionnaire Computer-assisted structured interview Midlife in the United States study (2004–2006; 2011–2014)	299 working unpaid carers	• Secondary workplace stressors had stronger associations vs primary caregiving stressors on wellbeing • Negative work-to-family spillover—lower life satisfaction and worse subjective wellbeing • Black working carers reported less work-to-family spillover, higher control/religious coping and more positive affect than white working carers		✓
[77]	Netherlands	Qualitative	Semi-structured focus group interviews	25 working unpaid carers of older adults	• Priorities of work, family and caregiving came before own health • Carers reported fatigue, anxiety, guilt, and no time for relaxation • Colleague support needed	✓	✓
[79]	Taiwan	Quantitative	Cross-sectional self-completed survey data	176 family carers of a person living with dementia	• When caregiving demands are high and interferes with work on a low level, health declines but only in those with average/low preparedness • Greater preparedness helps lower work/care conflict and role strain		✓
[78]	Canada	Quantitative	Cross-sectional data from 2012 Statistic Canada’s General Social Survey Cycle 26	2874 employed unpaid carers	• Flexible work environment—poor physical health less likely • No association found with mental health • Taking days off, reducing hours and turning down job offer negatively impacts health	✓	✓
[81]	Canada	Qualitative	Semi structured interviews	13 employed, unpaid carers	• Participants described combination as difficult to balance • Managing both was physically/mentally challenging • Effects are increased with time restraints	✓	✓
[83]	USA	Quantitative	Secondary analysis of baseline data from a longitudinal surveys (2017–2020)	90 family carers of home hospice cancer patients	• Employed carers – higher levels of burden and anxiety/depression • Employment moderates the impact of burden on global health • Among high-burden carers, those who worked had more positive wellbeing		✓
[84]	Canada	Quantitative	Cross-sectional analysis of 2018 Canadian General Social Survey	4291 carer-employees	• Work interferences due to caregiving significantly associated with mental health • Carer-friendly workplace policies—more positive mental health • Depression decreased with flexible working		✓

### Patient and Public Involvement

One carer as public advisor (JC) and one social care provider (PG) were part of the research team which helped to synthesise and interpret the evidence, and jointly develop the *Carers’ Work and Health Support Model*. In addition, we consulted six paid and unpaid carers about the model, who discussed how the first version of the model based on the existing literature mirrored their experiences, whilst also adding additional factors, which are identified in italics in the model, indicating the need for targeted research into these factors in particular.

## Results

### Overview of included studies

This scoping review included 58 studies from 21 countries (see Fig. 1 for PRISMA flowchart). Included studies were predominately conducted in the USA (*n* = 21), followed by seven studies in Canada and five studies from the UK. The remaining evidence was scattered across various countries; with a smaller number from the Netherlands and Australia (*n* = 3 each) and China, Japan, Sweden, and New Zealand (*n* = 2 each). Individual studies were included from Taiwan, Austria, Singapore, South Korea, Italy, Spain, Germany and Norway. Three studies were multi-country. One study included data from the UK and Belgium; a second from Puerto Rico, Mexico and Colombia; and a third drew data from 21 high-income European countries. In reference to methodology, the included studies contained a high number of quantitative methods (*n* = 50), followed by qualitative methods (*n* = 6) and a small number of mixed methods (*n* = 2). Table 1 provides an overview of study characteristics.

### Evidence into the relationship between paid work and health

**Fig. 2:**
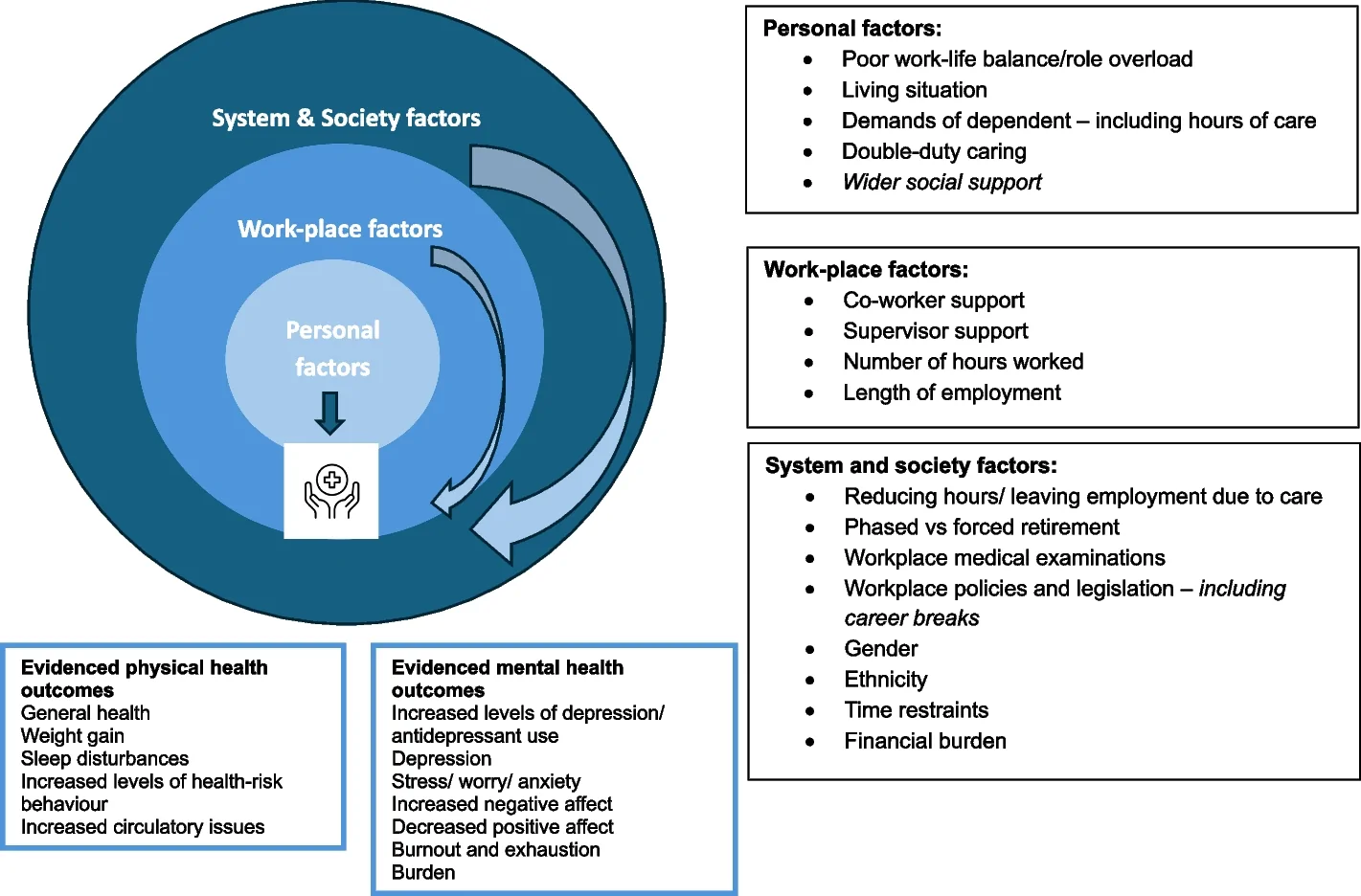
The Carers’ Work and Health Support Model for unpaid carers

Figure 2 shows a new model depicting the intricate relationship between work and health in unpaid carers – the *Carers’ Work and Health Support Model*. Factors that have been linked to physical and/or mental health and wellbeing have been categorised on macro, meso, and miso environmental levels, focusing on system and societal factors; work-place factors; and personal factors. Evidence from each factor is discussed below as to how it is found to be linked to physical and/or mental health in unpaid carers, with public consultation adding additional factors which were not evidenced in the included literature, including wider social support.

### Personal factors

Twenty studies evidenced personal factors to contribute to the relationship between paid work and health in unpaid carers, specifically focusing on role overload/poor work-life balance and demands of the dependant, as well as living situation. Thirteen studies showed how poor work-life balance and role overload negatively affected mental health, with less evidence on its relationship to physical health [25, 73, 79]. There is consensus in the literature including both cross-sectional and longitudinal quantitative studies and qualitative studies using semi-structured interviews, that providing unpaid care whilst working is linked to: increased levels of stress and emotional strain [1, 48, 73]; feeling moody and irritable [5], negative affect [13, 39], depression [32, 45]; and general mental health problems [46]. Focusing on employed unpaid carers of US cancer survivors for example, [32] reported that 38% (of *n* = 202) struggled to balance work and care, which was linked to depression and anxiety. In a similarly sized sample of carers of head and neck cancer patients in the US (*n* = 188), role captivity, defined as an intrapsychic feeling of entrapment within the care role, was linked to higher levels of distress [1]. However, one study evidenced how being employed whilst caring was linked to higher levels of quality of life than being unemployed, among a large sample (*n* = 1188) of unpaid carers for people with dementia in the UK [12].

Looking at the link between providing unpaid care and being in employment on physical health, three quantitative studies from the US and Taiwan, one of which also conducted focus groups with unpaid carers, evidenced how greater work-caring conflicts were significantly associated with poorer self-reported health [25, 73, 79].

Seven studies reported how the demands of the dependant, specifically the hours of unpaid care, affected carers’ health and employment. Similar to poor work-life balance, higher caring hours were linked to poorer mental health outcomes more often in comparison to physical health outcomes, because fewer studies focused on physical health. In a sample of over 21,000 employees in Sweden, some of whom were providing unpaid care, [63] reported that both groups providing either fewer than five hours, as well as providing more than five hours of unpaid carer per week, were statistically associated with sleep disturbances, and thus physical health, compared to no unpaid care.

Across all other included studies focusing on hours of care and demands of the dependant, including evidence from Canada, the UK and US, higher care demands, including intensive caregiving of 80 + hours a week [42], were statistically and qualitatively linked to greater stress, depression, and anxiety [16, 42, 43, 64]. Interestingly, when comparing unpaid carers who were in employment with those caring for their relative full-time, the former fared better when care burden was high in a sample of carers with dependants in home hospice cancer care [83]. Whilst employed carers overall experienced higher levels of burden, those who worked experienced significantly higher levels of well-being as measured on the Positive Affect and Wellbeing Scale, compared to non-employed carers [83]. Evidence from Japan further corroborates how caring demands in general (details of caring hours were not provided), were found to be a risk factor for depression in a mixed-gender sample of over 700 workers in total [34].

One study evidenced how living situation affected the relationship between work and mental wellbeing in unpaid carers. In a large sample of Canadian unpaid carers who were employed, [20] reported how both physical and emotional strain were highest among those who lived with the dependant, compared to those with dependants living both within one hour, or more than one hour’s journey away.

Being employed in the care sector, and thus facing double-duty caring, was found to cause stress and anxiety in four New Zealand unpaid/paid carers [19]. However, the evidence is very limited not only in terms of number of studies but also inadequate sample sizes, thus more, larger, studies are required.

### Work-place factors

Fifteen studies reported on the influence of work-place related factors on the relationship between health and employment. A further nine studies illustrated additional work-related evidence, without fitting into a specific factor. In the workplace, supervisor and managerial support and co-worker support have been evidenced to be key factors in affecting work-related health and well-being when providing unpaid care in particular. Having a supportive supervisor was found to: ease emotional strain [72] and be linked to work-life balance [33]; but conversely was linked to increased depressive symptoms and decreased life satisfaction where family to work conflict was high and supervisor support low [45]. Regardless of the type of manager, in a survey of over 360 full-time employed unpaid carers in the US, [73] found that disclosure of caregiving to employers was linked to increased emotional and physical strain, with workplace policies not significantly buffering the carer-work conflict. However, quantitative and qualitative evidence from the Netherlands, UK, and the US showed the positive contributions and value of co-worker support on employed unpaid carers’ well-being [5, 33, 72, 77]. Specifically, supportive colleagues could lead to reduced emotional strain when trying to manage caring with work and positive impact well-being [72].

Seven quantitative studies from China, the US, South Korea, Germany, and Australia reported how the numbers of hours worked affected unpaid carers’ well-being and physical health. Working by the hour on zero-hour contracts was statistically linked to poorer physical and mental health compared to employed carers with substantive contracts [25]. Looking at both the numbers of hours worked and unpaid care provided, [11] evidenced how female employees in China (overall n = 10,800 +) who worked both 50 + hours and provided 20 + hours of care a week had the poorest health outcomes, with increased risk of chronic disease and being under/overweight. No other research has evidenced weight issues as work- and caring-related health outcomes, whilst working and caring longer hours has been linked to poorer psychological wellbeing [59]. In contrast, [3] reported no link between working hours and stress, except for carers supporting dependants with a mental disability, who experienced reduced levels of stress with higher working hours. This is corroborated by quantitative findings from Germany, the UK and the US, with full-time employed unpaid carers or those with higher levels of employment experiencing better mental health [67] and decreased levels of distress [1] and being less likely to be prescribed antidepressants, compared to part-time or unemployed high-intensity carers [17].

Only one study focused on length of employment, with unpaid carers found to experience higher levels of burnout with longer employment duration in the University sector [37].

Additional evidence does not fit into a specific category of factors but focused on the wider overarching relationship between paid work and care. Evidence was mixed as to whether physical and/or mental health was better in working or not working unpaid carers [2, 22, 52, 56, 57, 68]. O’Neill et al.[57], Socci et al. [68], and Kyo et al [41] reported non-working carers to experience poorer physical health as well as mental health and/or levels of burden, although [41] also reported the opposite for mental health. Similarly, [56] evidenced that working carers, as opposed to non-employed carers, had better physical health and fewer mobility problems. Findings by [41] in the US regarding poorer mental health in working carers were corroborated by [2] in New Zealand. All studies were quantitative and included varying samples between 132 and over 1000 unpaid carers, utilising standardised tests for mental health and physical functioning. Whilst the tests used differed, and analyses often took into account other potentially mediating factors such as age and gender or socio-economic background, the relationship between working, caring and health outcomes is broad, encompassing various moderating factors which some studies have not included. Interestingly, when comparing stress in the workplace with that from caring, [74] showed that work-place stressors had a stronger association with poorer wellbeing than stressors resulting from providing unpaid care. This suggests the importance of focusing on work-place environment issues to support a healthy workforce who has unpaid caring duties at home.

### System and Societal factors

Twenty-seven studies evidenced systemic or societal factors which influence the relationship between paid work and health in those who provide unpaid care. Seven studies evidenced how reducing hours or leaving employment due to caring duties was linked to poorer mental wellbeing, including higher levels of anxiety and depression [1, 5, 28, 46, 49, 54, 58, 78]. The only study reporting mixed findings was qualitative and reported how reducing hours could support positive impacts on health and wellbeing if it created a more manageable care situation [5]. However, the picture was mixed, with some experiencing poorer health and wellbeing, corroborating the quantitative evidence. The only study looking at changes to working hours reported how no changes to working hours, versus being forced to reduce hours, was linked to better physical health [68].

The issue of phased vs. forced retirement is also a means to reduced working hours. In a large survey of over 1600 employed unpaid carers from 680 different organisations in the Netherlands, [29] showed that perceived access to phased retirement was linked to lower stress levels. Perceived access to workplace medical examinations to check up on carers’ health was also linked to reduced stress levels.

Five studies reported how workplace policies and legislation affected unpaid carers’ wellbeing. Whilst flexible working was found to be impacting positively on carers’ mental wellbeing [33, 78, 84], taking time off or reducing hours was evidenced to impact negatively on wellbeing [58, 78]. Shift patterns affecting health outcomes were evidenced by [60], with US unpaid carers working full-time and providing care between late shifts experiencing poorer mental wellbeing than employed unpaid carers providing care on their days off.

Gender influences the relationship between work and health and affects health outcomes differently, as evidenced in eight studies [7, 17, 18, 24, 30, 38, 47, 71]. All evidence was quantitative and compared male and female carers with different degrees of employment status, mostly reporting worse health/mental health outcomes for female carers, sometimes depending on the level of employment or years of follow-up (i.e. [24, 47]. In an Australian longitudinal survey of over 400 carers and 400 + matched control non-carers, female carers in full-time employment aged between 40–64 experienced significant reductions in physical and mental health after two years of caring, whilst male carers in that age bracket who were not in paid work were found to experience an improvement in these outcomes. The finding of female carers faring worse than male carers is repeated in research from other countries. [7] reported how women were more likely to experience poor mental health when working and caring compared to men, whilst [18], using data from over 20,000 individuals in China, showed how supporting spouses with instrumental activities of daily living, such as preparing a meal and managing finances, was linked to higher levels of depression in female carers working full-time, but not male carers. Two studies reported how working part-time as opposed to full time was linked to poorer mental well-being for female carers, whilst the level of employment did not seem to affect the impact of caring on well-being in men [17, 30].

Only one study evidenced how ethnicity affected the relationship between work and health. Compared to white working carers, black working carers in the US reported higher religious or spiritually facilitated coping and wellbeing [74].

Financial burden was another evidenced factor in the relationship between work and health, reported in five studies from the US and Singapore. Decreased income, increased debt, as well as stopping working, were more likely to occur among unpaid carers from low-income households caring for someone with cancer compared to those caring for someone with a different problem, but associations with mental health were mixed or non-significant [6]. Making work changes as a result of unpaid caring was linked to greater likelihood of financial burden in a different study among carers of African American cancer survivors as well with consequent increased levels of anxiety [32]. Rather than finding directly linked financial burden with mental health outcomes, [39] showed how perceived financial need moderated the relationship between work-caregiving conflict and psychological wellbeing. Aligned with this study, [48] found that greater financial strain at baseline in unpaid carers for someone with dementia was linked to higher levels of role overload over time.

To alleviate the caring demands, unpaid carers can employ paid carers, potentially introducing, or adding to existing, financial burden. However, only one study [50], including employed unpaid carers, found that employing a paid carer was mentally beneficial, stressing the need for further research.

### Summary of impacts on mental and physical health

As can be clearly seen in Table 1, the majority of included studies have evidenced impacts of paid work and unpaid caregiving on mental health (n = 52 studies), with fewer studies having focused on physical health outcomes (n = 21 studies), while 16 explored both mental and physical health outcomes. Mental health outcomes included depression, anxiety, stress, burnout, and burden, whilst physical health outcomes most often included general physical health but also some specific issues such as weight gain and sleep disturbances (also see Fig. 2 for an overview of physical and mental health outcomes). Thus, evidence into the different impacts on physical health remains more limited.

## Discussion

This is the first scoping review to systematically synthesise the evidence into the relationship between paid work and health in unpaid carers, comprising evidence from 21 countries. We propose a new model, based on the identified international evidence and additional public stakeholder consultations – the *Carers’ Work and Health Support Model*. This novel model, the first to link work with health for this population, clearly identifies factors on a personal, workplace, and system and societal level that affect unpaid carers’ physical and mental health, including weight gain, sleep disturbances, anxiety, depression, and burnout.

Overall, there is strong evidence on the impact of unpaid caring on mental health whilst working. Various factors have been linked to poorer mental health outcomes, including burnout, depression, anxiety, increased stress levels, and general carer burden [32, 37]. Working full-time as opposed to part-time or having zero-hour contracts for example was considered overall beneficial [67], whilst having to reduce working hours or being forced into retirement as a result of caring duties was found to have negative impacts on carers’ mental health [54]. Burnout can be a concerning determinant of poor physical and mental health, and is not restricted to work-related factors. A recent annual report by Mental Health UK 51 showed that one in five adults of over 4,000 sampled across the country had to take time off work in the past year due to burnout. Considering that the included evidence in this review has studied unpaid carers in different jobs and work settings, the *Carers’ Work and Health Support Model* is relevant regardless of job sector – as unpaid caring duties do mostly negatively impact people’s mental health when combined with employment.

In light of the demographic make-up of the unpaid carer population – which is predominantly female [82] – this review is the first to collectively show that women who provide care and are in employment appear to have poorer mental health outcomes than male unpaid carers who are also in employment (i.e. [7, 18, 71]. The included studies did not point to a particular reason as to why this is the case. One possible reason may be that women are expected to provide more unpaid care hours, regardless of having a job or not, than men, which leads to higher levels of time demands on female carers in employment. There are numerous reports of gender norms and hours providing unpaid care (i.e. [65]. It is important to highlight this inequality, especially as the majority of unpaid carers who are in employment are in their 50 s and 60 s (WHO, 2022), a time when women go or have gone through menopause and may experience other gender-specific health issues [53]. Therefore, it is crucial for female unpaid carers in particular to be supported in the workplace and ensure a good work-life balance, which regardless of gender has been found to negatively affect depression and other mental health aspects when not available [32, 45]. Taking all these into account, women are disproportionately affected worse than men with caring duties and in employment, and work-place interventions to support the unpaid care workforce remain healthy and well in employment may need to take a gender-specific approach.

Besides gender, little focus has been paid to individual carer characteristics and how these may influence the relationship between work and health. For example, it is important to consider carers not as a homogenous group, as carers have different characteristics and associated needs. For example, there was no clear distinction between carers co-residing or living separately to the person they cared for. This is an important carer characteristic, as living separately can increase the time commitment to caring, including travel time or having to prepare separate meals than in the carer’s own household, and increase emotional demands [80]. Moreover, it is important to consider carers providing care for both younger children and older relatives. Evidence suggests that more sandwich-generation carers report financial and emotional difficulties compared to non-sandwich carers who only care for one individual [44]. Future research should therefore consider the heterogenous nature of unpaid carers in more depth and how their characteristics and types of caring roles may affect the relationship between work and health.

Compared to mental health, fewer included studies evidenced impacts on unpaid carers’ physical health when in employment. However, evidence does indicate that working whilst caring amplifies stress (mental health), and reduces personal time to do previously enjoyed activities, leading to a time deficit which in many carers is reflected in sleep disturbances [63] and lack of sleep as well as weight gain [11]. The latter is likely to be affected by less time to cook healthy meals and over-reliance on quick, potentially unhealthy, foods, albeit further research is required to establish the reasons Whilst unpaid care can involve heavier personal caring duties including lifting [14], it appears that the dual nature of effectively two jobs – a paid job and an unpaid caring job – is linked to greater impacts on mental health than physical health. In contrast, physical health was more or less similarly affected in paid carers ([26] submitted). This may be the result of expected heavy lifting and transferring of clients in the adult social care workforce, whereas unpaid carers could, if financially feasible, employ a paid carer to engage in these caring duties.

One possible reason why evidence painted a complex picture of the relationship between work and health for unpaid carers could be differences in legislation and welfare regimes. Different countries have different rules and regulations, taxation, social spending, carers leave entitlements, and variations in the social care sector and how this is funded and governed. Focusing on Europe for example, welfare is generally compared alongside four types of European social models – Scandinavian, Anglo-Saxon, Continental, and Southern [66]. These differences may affect whether unpaid carers need to continue working whilst caring, or whether paid carers how up to work ill instead of taking legitimate time off to get better.

Whilst evidence included in this review is very comprehensive, featuring 58 studies from 21 countries, it is important to stress that a substantial majority of the evidence was generated in high-income, mostly Western, countries. No evidence emerged from lower-income countries, and only one study referenced middle-income countries (LMICs) [52], where unpaid carers can face substantially different challenges to those in high-income countries. The *Carers’ Work and Health Support Model* is therefore currently Western-influenced and may need to be adapted or sense-checked with evidence from LMIC contexts. For example, recent research into the gender gap in unpaid care in Colombia has shown that women remain the primary caregivers and are thus often not participating in the labour market [4], which is also evidenced in other LIMCs such as Ghana [55] and India [36]. Therefore, future research needs to explore the *Carers’ Work and Health Support Model* in different cultural and systemic contexts.

Focusing on possible solutions to negative impacts of work on health, one of the more frequently evidenced factors impacting unpaid carers’ mental health and well-being is supportive management and co-workers [5, 72]. This is mirroring established knowledge in occupational psychology and health [23, 31] in general for the workforce. Given the substantial impact of positive and supportive work-environment on a personal level, any interventions to support unpaid carers to stay well in employment may need to take an organisational angle involving coworkers and management. Existing interventions educating managers about mental health and thus being able to support their employees better have been beneficial [23].

### Limitations

Whilst this scoping review benefitted from following PRISMA guidance for scoping reviews [76] and comprised over 50 relevant published studies from 21 countries, with a spread of quantitative and qualitative research and different types of methodologies and varied sample size, including large national datasets, there were some limitations to the included evidence. First, some quantitative studies failed to include control groups, which made it difficult to compare issues in unpaid carers who were employed compared to employees without caring duties for example. Second, only on study appeared to have been published in LMICs – in three upper middle-income countries to be precise [52]. Whilst this may be the case due to the inclusion of English or German language articles only, based on the languages spoken of the research team, most research from LMICs is published in English, consequently either that evidence has not been gathered or published, or not in international journals likely to be included in the databases we searched.. To ensure a more representative understanding of the relationship between paid work and health in unpaid carers, it is therefore important to conduct research in LMICs to supplement the ample evidence from high income countries, or explore alternative ways to uncover existing evidence. Third, factors such as double-duty caring, living situation, and ethnicity, which have only been evidenced in one study each, require further research.

## Conclusions

Providing unpaid care whilst also holding down a job can be challenging and can affect both mental and physical health. Unpaid carers working full-time hours and being in control of their hours, with supportive management and colleagues, experience overall better work-related health outcomes. Whilst most evidence concerned mental health outcomes, there is relatively sparse research into the impacts of working whilst caring on physical health outcomes. Given the growing population of unpaid carers, who can oftentimes financially suffer if they have to leave employment to provide full-time care [32], it is critical to find effective solutions to maintain a healthy workforce with unpaid caring duties. Overall, findings indicate avenues for solutions of possible interventions to support employed unpaid carers better. These need to be co-produced and tested. The *Carers’ Work and Health Support Model for unpaid carers* also needs to be sense-checked with evidence from employed unpaid carers residing in LMICs where support infrastructures for carers can greatly differ.

## Data Availability

No datasets were generated or analysed during the current study.

## Appendix 1

### PRISMA extension for scoping reviews checklist

**Table:**

Section	Item	PRISMA-ScR checklist item	REPORTED ON PAGE #
Title			
Title	1	Identify the report as a scoping review	1
Abstract			
Structured summary	2	Provide a structured summary that includes (as applicable): background, objectives, eligibility criteria, sources of evidence, charting methods, results, and conclusions that relate to the review questions and objectives	1
Introduction			
Rationale	3	Describe the rationale for the review in the context of what is already known. Explain why the review questions/objectives lend themselves to a scoping review approach	2
Objectives	4	Provide an explicit statement of the questions and objectives being addressed with reference to their key elements (e.g., population or participants, concepts, and context) or other relevant key elements used to conceptualize the review questions and/or objectives	3
Methods			
Protocol and registration	5	Indicate whether a review protocol exists; state if and where it can be accessed (e.g., a Web address); and if available, provide registration information, including the registration number	n/a
Eligibility criteria	6	Specify characteristics of the sources of evidence used as eligibility criteria (e.g., years considered, language, and publication status), and provide a rationale	3
Information sources*	7	Describe all information sources in the search (e.g., databases with dates of coverage and contact with authors to identify additional sources), as well as the date the most recent search was executed	4
Search	8	Present the full electronic search strategy for at least 1 database, including any limits used, such that it could be repeated	4
Selection of sources of evidence†	9	State the process for selecting sources of evidence (i.e., screening and eligibility) included in the scoping review	4
Data charting process‡	10	Describe the methods of charting data from the included sources of evidence (e.g., calibrated forms or forms that have been tested by the team before their use, and whether data charting was done independently or in duplicate) and any processes for obtaining and confirming data from investigators	4
Data items	11	List and define all variables for which data were sought and any assumptions and simplifications made	n/a
Critical appraisal of individual sources of evidence§	12	If done, provide a rationale for conducting a critical appraisal of included sources of evidence; describe the methods used and how this information was used in any data synthesis (if appropriate)	n/a
Synthesis of results	13	Describe the methods of handling and summarizing the data that were charted	4
Results			
Selection of sources of evidence	14	Give numbers of sources of evidence screened, assessed for eligibility, and included in the review, with reasons for exclusions at each stage, ideally using a flow diagram	4
Characteristics of sources of evidence	15	For each source of evidence, present characteristics for which data were charted and provide the citations	4
Critical appraisal within sources of evidence	16	If done, present data on critical appraisal of included sources of evidence (see item 12)	n/a
Results of individual sources of evidence	17	For each included source of evidence, present the relevant data that were charted that relate to the review questions and objectives	n/a
Synthesis of results	18	Summarize and/or present the charting results as they relate to the review questions and objectives	5–10
Discussion			
Summary of evidence	19	Summarize the main results (including an overview of concepts, themes, and types of evidence available), link to the review questions and objectives, and consider the relevance to key groups	11
Limitations	20	Discuss the limitations of the scoping review process	12
Conclusions	21	Provide a general interpretation of the results with respect to the review questions and objectives, as well as potential implications and/or next steps	12
Funding			
Funding	22	Describe sources of funding for the included sources of evidence, as well as sources of funding for the scoping review. Describe the role of the funders of the scoping review	13
